# Comparisons of six endoscopy independent scoring systems for the prediction of clinical outcomes for elderly and younger patients with upper gastrointestinal bleeding

**DOI:** 10.1186/s12876-022-02266-1

**Published:** 2022-04-13

**Authors:** Yajie Li, Qin Lu, Mingyang Song, Kexuan Wu, Xilong Ou

**Affiliations:** 1 Department of Gerontology, Zhongda Hospital, School of Medicine, Southeast University, Nanjing, Jiangsu 210009 People’s Republic of China; 2 Department of Gastroenterology, Zhongda Hospital, School of Medicine, Southeast University, Nanjing, Jiangsu 210009 People’s Republic of China

**Keywords:** Upper gastrointestinal bleeding (UGIB), Elderly patients, Comparision, Scoring system, ROC curve

## Abstract

**Objectives:**

To compare the predictive ability of six pre-endoscopic scoring systems (ABC, AIMS65, GBS, MAP(ASH), pRS, and T-score) for outcomes of upper gastrointestinal bleeding (UGIB) in elderly and younger patients.

**Methods:**

A retrospective study of 1260 patients, including 530 elderly patients (age $\ge$ 65) and 730 younger patients (age < 65) presenting with UGIB, was performed at Zhongda Hospital Southeast University, from January 2015 to December 2020. Six scoring systems were used.

**Results:**

ABC had the largest areas under the curve (AUCs) of 0.827 (0.792–0.858), and 0.958 (0.929–0.987) for elderly and younger groups for predicting mortality respectively. The differences of the AUCs for predicting the outcome of mortality and rebleeding between the two groups were significant for ABC and pRS (p < 0.01). For intervention prediction, significant differences were observed only for pRS [AUC 0.623 (0.578–0.669) vs. 0.699 (0.646–0.752)] (p < 0.05) between the two groups. For intensive care unit (ICU) admission, the AUC for MAP (ASH) [0.791 (0.718–0.865) vs. 0.891 (0.831–0.950)] and pRS [0.610 (0.514–0.706) vs. 0.891 (0.699–0.865)] were more effective for the younger group (p < 0.05 and p < 0.01, respectively). For comparison of scoring systems in the same cohort, ABC was significantly higher than pRS: AUC 0.710 (0.699–0.853, p < 0.05) and T-score 0.670 (0.628–0.710, p < 0.01) for predicting mortality in the elderly group. In the younger group, ABC was significantly higher than GBS and T-score (p < 0.01). MAP(ASH) performs the best in predicting intervention in both groups.

**Conclusions:**

ABC and pRS are more accurate for predicting mortality and rebleeding in the younger cohort, and pRS may not be suitable for elderly patients. There was no difference between the two study populations for GBS, AIMS65, and T-score. Except for ICU admission, MAP(ASH) showed fair accuracy for both cohorts.

## Introduction

Upper gastrointestinal bleeding (UGIB) is a common medical emergency. The incidence of morbidity has been reported at 48–160 per 100,000 adults annually [1], and the mortality rates range from 2 to 8% [2]. UGIB accounts for 300,000 hospitalizations annually with an economic burden of $3.3 billion [3]. The highest incidence of acute UGIB is in elderly patients, with about 1% of patients aged 80 years hospitalized due to an acute UGIB attack [4].

The international consensus suggests that UGIB be managed using "early risk stratification" with valid prognostic indicators [5]. Risk assessment score systems that include pre-endoscopy and post-endoscopy scales have been developed to predict clinically relevant outcomes [6]. Studies showed that these scoring systems distinguish low-risk patients who can be potentially managed as outpatients, thereby allowing more efficient use of resources [7]. Another study suggested that these score systems distinguish patients at higher risk who might require emergency endoscopy or management in an ICU; the Rockall score and Progetto Nazionale emorragia digestive score require endoscopy before calculation [8]. However, requiring endoscopy might delay risk assessment in some healthcare units, as there can be considerable delays in performing an endoscopy outside of working hours or on weekends [9]. Some patients cannot tolerate endoscopy. Therefore, much attention has been paid to pre-endoscopic scoring systems for UGIB, calculated soon after admission. The most widely used and validated score systems are the pre-endoscopic Rockall score (pRS), the Glasgow Blatchford Score (GBS), and AIMS65. A systematic review of 16 studies concluded that the GBS has higher sensitivity and specificity to predict hospital-based intervention and 30-day mortality requirements than RS and AIMS65 [10]. However, other studies showed that the GBS accurately predicts patients who will require intervention; while, its prediction of mortality is relatively poor [11]; When it comes to predicting mortality, AIM65 does better than GBS and pRS; however, the area under the receiver operator characteristics curve (ROC of AUCs) are generally no higher than 0.80, suggesting that the clinical application of predicting this endpoint is limited [7].

T-score is another pre-endoscopic score system that appears to predict high-risk endoscopic stigmata, mortality, and rebleeding [12]. Recently, several new scoring systems have been developed, including the MAP(ASH) and ABC scores [13, 14]; however, their accuracies need to be verified. Recent guidelines suggested using risk scores for patients with UGIB; according to the guidelines, the scores should be used to identify and treat high-risk patients; however, their precise role in practice (especially for a daily growing number of elderly patients) remains uncertain [15]. Therefore, this retrospective study aimed to evaluate the effectiveness of six pre-endoscopic risk assessment scores in predicting mortality, intervention, rebleeding, and ICU admission from UGIB in elderly and younger patients.

## Methods

### Study design

A retrospective cohort study was conducted at Zhongda Hospital Affiliated to Southeast University from January 2015 to December 2020. The predetermined clinical endpoints were the composite endpoint of need for hospital-based interventions, including blood transfusion, endoscopic treatment, interventional radiology, and surgery or death.

UGIB was defined as bleeding from the upper gastrointestinal tract characterized by coffee-ground vomiting, hematemesis, or melena [16, 17]. Variceal and non-variceal UGIB were included in the analysis. Most UGIB patients underwent endoscopy. Only a few patients with poor general conditions who did not undergo endoscopy were excluded. The on-duty gastroenterologist determined the timing of endoscopy and whether to perform endoscopic therapy.

Rebleeding was defined as the presentation of fresh hematemesis and/or melena associated with the development of shock (pulse > 100 beats/minute and/or systolic blood pressure < 100 mmHg) or decreased hemoglobin concentration by more than 2 g/dL after successful initial treatment. Rebleeding included cases requiring a second endoscopy therapy, interventional radiology, or surgery [18]. The indications for blood transfusion were hemoglobin levels falling to < 7 g/dL in average patients or < 8 g/dL in patients with a high risk of heart disease [16, 17].

Endoscopic therapy included injection of diluted epinephrine, clipping, or thermal captive coagulation. Variceal hemorrhage was treated by transjugular intrahepatic portosystemic shunt, band ligation, or injection of tissue glue.

### Data collection

Patients who presented with hematemesis, coffee-ground vomiting, or melena were included in the analysis. Older adults were defined as aged $\ge$ 65 years. Patients with primary diagnoses other than UGIB were excluded.

We recorded demographic data (age and sex), clinical presentation, mental state, comorbidities (diabetes, hypertension, cardiac diseases, liver disease, chronic pulmonary diseases, cerebral infarction, renal disease or disseminated malignancy), medications history (including nonsteroidal anti-inflammatory drugs, antiplatelet drugs or oral anticoagulants), hemodynamic parameters (pulse rate and blood pressure), hemoglobin, biochemical parameters (albumin, creatinine, blood urea nitrogen and coagulation panel). Other parameters analyzed were needed for the blood transfusion, endoscopic treatment, interventional radiology, or surgery. The Clinical outcomes documented were rebleeding, interventions including endoscopic treatment, transfusion, radiologically guided hemostasis, or surgery, ICU admission, and 30-day mortality. The data were used to calculate each patient's ABC score, MAP(ASH) score, GBS, T-score, pRS, and AIMS65 scores (Table 1).

**Table 1:** Components of the AIMS65, pRS, T-score, MAP, GBS, ABC

AIMS65	Score	GBS		Score	
Albumin < 3.0 mg/dl	1	Blood urea, mmol/L			
INR > 1.5	1	6.5–8		2	
GCS < 14	1	8–10		3	
SBP < 90 mmHg	1	10–25		4	
Age > 65 yrs	1	> 25		6	
Maximum score	5	Hemoglobin, g/dl, men			
pRS		men	women		
Age		12- < 13		1	0
< 60 yrs	0	10- < 12	10- < 12	3	1
60–79 yrs	1	< 10	< 10	6	6
> 80 yrs	2	SBP, mmHg			
Shock		100–109		1	
No shock	0	90–99		2	
Pluse > 100, SBP > 100 mmHg	1	< 90		3	
SBP < 100 mmHg	2	Pluse (> = 100/bpm)		1	
Comorbidity		Melena		1	
No major	0	syncope		2	
CHF, IHD, or major comorbidity	2	Liver disease		2	
Renal failure, liver failure, metastatic cance	3	Heart failure		2	
Maximum score	7	Maximum score		23	

T-score		ABC	
General conditions		Age	
Poor	1	60-74yrs	1
Intermediate	2	≥ 75yrs	2
Good	3	Blood tests	
Pulse (beats /min)		Urea > 10 mmol/L	1
> 110	1	Albumin < 30 g/L	2
90–110	2	Creatinine	
< 90	3	100-150 μmol/L	1
SBP, mmHg		> 150 μmol/L	2
< 90	1	Comorbidity	
90–110	2	Altered mental status	2
> 110	3	Liver cirrhosis	2
Hemoglobin, g/dl		Disseminated malignancy	4
< 9	1	ASA score	
9–10	2	3	1
> 10	3	≥ 4	3
Maximum score	12	Maximum score	18
MAP			
M: altered mental status (Glasgow < 15)	1	A: albumin < 2.5 g/dL	2
A: ASAscore > 2	1	S: SBP < 90 mmHg	2
P (pulse): HR > 100	1	H: hemoglobin < 10 g/L	2
		Maximum score	9

GCS Glasgow Coma Scale, SBP Systolic blood pressure, CHF Congestive heart failure, IHD Ischemic heart disease, ASA: American Society of Anesthesiologists

### Data analysis

We use MedCalc version 19 for statistical calculations. Mean $\pm$ standard deviation was calculated for descriptive statistics. The receiver-operating curve(ROC) was used for assessing the prognostic value of each scoring system, the area under the curves (AUCs) of the six scoring systems were calculated one by one for mortality, intervention, ICU admission, and rebleeding. While 0.5 < AUCs ≤ 0.7, 0.7 < AUCs ≤ 0.9, and AUCs > 0.9 represent poor, fair and good accuracy, respectively. Then Delong test was used in achieving the comparison of different AUCs among the six score systems. A p-value < 0.05 indicates statistically significant.

## Results

### Study population

A total of 1489 patients were enrolled, of which 1260 (84.6%) patients were finally analyzed. Of these, 229 (15.4%) patients were excluded from the study for the reasons as follows: 123 patients did not have sufficient data for the study; 106 patients did not undergo endoscopy. The median age was 54.8 (range 18–89 years). They were divided into two groups (Table 2): the elderly group (65–89 years, mean age 72.9 ± 6.1) and the younger group (18–64 years, mean age 48.7 ± 12.2). Of the 530 elderly patients, 44 patients (8.3%) died in 30 days, 240 (45.3%) required intervention, and 112 (21.1%) patients suffered from rebleeding. In the control group, among 730 younger patients, 24 (3.3%) died within 30 days, 304 (41.6%) required intervention, and 60 (8.2%) patients suffered from rebleeding. UGIB was more common in men than women, and the trend was more pronounced in the younger group. Statistical significance was observed between the two groups concerning the differences in mortality and rebleeding, while the intervention difference between the elderly and younger UGIB patients was insignificant.

**Table 2:** Characteristics of the patients

Variables	Elderly patients	Younger patients	P-value (p < 0.05)
Age	72.9 $\pm$ 6.1	48.7 $\pm$ 12.2	–
Sex (male/female)	350:180	573:157	< 0.01
Comorbidity			
Cirrhosis	52 (9.8%)	108(14.8%)	< 0.01
Renal failure	56 (10.6%)	10(1.4%)	< 0.01
Any malignancy	46 (8.7%)	118(16.2%)	< 0.01
PCI	52 (9.8%)	28(3.8%)	< 0.01
Heart failure	16 (3.0%)	2(0.3%)	< 0.01
Hypertension	294 (55.5%)	212(29%)	< 0.01
Diabetes	102 (19.2%)	90(12.3%)	< 0.01
Chronic lung disease	18 (3.4%)	2(0.3%)	< 0.01
Medications			
NSAIDs	6 (1.1%)	4(0.5%)	> 0.05
Aspirin	130 (24.5%)	62(8.5%)	< 0.01
Clopidogrel	64 (12.1%)	20(2.7%)	< 0.01
Oral anticoagulants	22 (4.2%)	4(0.5%)	< 0.01
Steroids	8 (1.5%)	6(0.8%)	> 0.05
Relevant variables and scores components (median (IQR))			
Systolic blood pressure(mmHg)	124(28)	120(24)	–
Pulse(beats/min)	79(18)	82(22.5)	–
Creatinine(μmol/L)	82(41)	73(27)	–
Hemoglobin(g/L)	89(43)	102.5(45)	–
Albumin (g/L)	33.7(8.45)	37.25(9.08)	–
Urea(mmol/L)	9.7(10.1)	7.9(6.35)	–
ASA score	3(1)	2(2)	–
Findings at endoscopy			
Duodenal/gastric ulcer	246 (46.4%)	424(58%)	< 0.01
Erosions	44 (8.3%)	56(7.7%)	> 0.05
Upper GI cancer	70 (13.2%)	28(3.8%)	< 0.01
Variceal bleeding	44 (8.3%)	88(12.1%)	< 0.05
Esophagitis	16 (3.0%)	10(1.4%)	< 0.05
Mallory-Weiss syndrome	16 (3.0%)	32(4.4%)	> 0.05
Normal	94(16.9%)	92(12.3%)	< 0.05
Outcomes			
Death (total)	44 (8.3%)	24(3.3%)	< 0.01
Intervention	240 (45.3%)	304 (41.6%)	> 0.05
Rebleeding	112 (21.1%)	60 (8.2%)	< 0.01

PCI percutaneous coronary intervention, NSAIDs nonsteroidal anti-inflammatory drugs, IQR interquartile range

### Comparison between the groups

#### Mortality

**Fig. 1:**
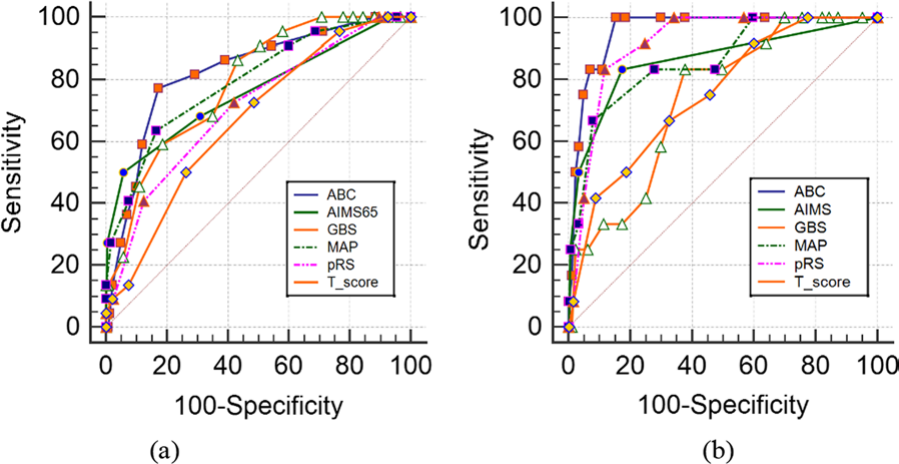
ROC curves for six scoring systems in evaluation of mortality **a** Elderly group **b** younger group

The comparisons of the six scoring systems for predicting mortality groups are displayed in Table 3. For both groups, AIMS65, GBS, MAP(ASH), and T-score had similar effectiveness (p > 0.05). By contrast, the accuracy of ABC and pRS for predicting mortality for the younger group was significantly higher than for the elderly group (p < 0.01). The ROC curves of the six scoring systems for predicting mortality for elderly and younger UGIB patients are shown in Fig. 1a, b, respectively.

**Table 3:** Comparisons of ROC curves for six scoring systems in the prediction of mortality between the two groups

Scoring systems	AUC (95%CI)	Sensitivity %(95%CI)	Specificity %(95%CI)	P-value
Elderly group/younger group				
ABC	0.827(0.763–0.890)/ 0.958(0.929 -0.987)	77.27(62.2–88.5)/ 100.00(73.5–100)	82.72(79.1–86.0)/ 84.66(80.5–88.3)	< 0.01
AIMS65	0.762(0.680–0.843)/ 0.862(0.740—0.983)	50.00(34.6–65.4)/ 83.33(51.6–97.9)	94.26(91.8–96.2)/ 82.67(78.3–86.5)	0.18
GBS	0.787(0.726 -0.848)/ 0.737(0.614—0.860)	86.36(72.6–94.8)/ 91.67(61.5–99.8)	56.79(52.3–61.2)/ 36.08(31.1–41.3)	0.67
MAP(ASH)	0.795(0.725 -0.864)/ 0.859(0.748 -0.970)	63.64(47.8–77.6)/ 66.67(34.9–90.1)	83.54(79.9–86.7)/ 92.05(88.7–94.6)	0.34
pRS	0.710(0.634 -0.786)/ 0.913(0.864—0.963)	72.73(57.2–85.0)/ 83.33(51.6–97.9)	58.02(53.5–62.5)/ 88.5(84.5–91.5)	< 0.01
T-score	0.670(0.596 -0.745)/ 0.749(0.615—0.883)	72.73(57.2–85.0)/ 75.00(42.8–94.5)	51.44(46.9–56.0)/ 54.26(48.9–59.6)	0.32

#### Intervention

**Fig. 2:**
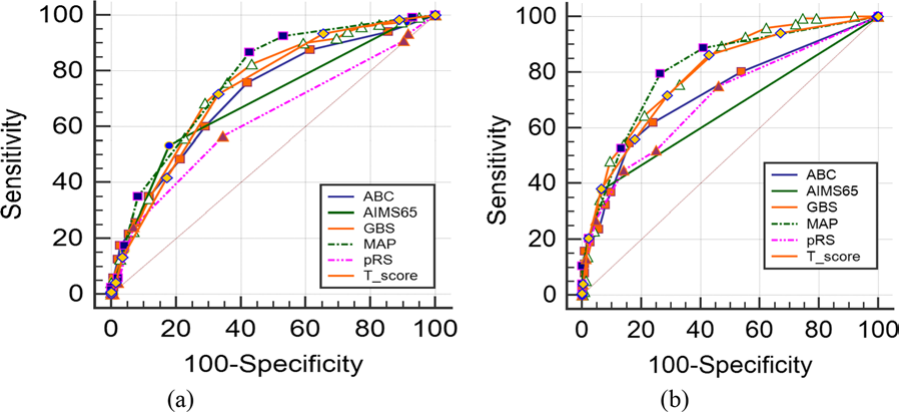
ROC curves for six scoring systems in evaluation of intervention **a** Elderly group **b** younger group

The comparisons of the six scoring systems for intervention prediction are displayed in Table 4. The AUC of AIMS65 for the elderly group was insignificantly greater than for the younger group (p = 0.25), while the other five scoring systems had larger AUCs for the younger group. Except for pRS (p < 0.01), the differences for evaluation of intervention between the groups according to the other four systems were insignificant (p = 0.73 for ABC, p = 0.07 for GBS, p = 0.16 for MAP(ASH), and p = 0.09 for T-score, respectively). The ROC curves for the two groups are depicted in Fig. 2a, b, respectively.

**Table 4:** Comparisons of ROC curves for six scoring systems in the prediction of intervention between the two groups

Scoring systems	AUC (95%CI)	Sensitivity %(95%CI)	Specificity %(95%CI)	P-value
Elderly group/ younger group				
ABC	0.713(0.670–0.757)/ 0.725(0.673–0.778)	75.83(69.9–81.1)/ 54.61(46.3–62.7)	57.93(52.0–63.7)/ 83.96(78.3–88.6)	0.73
AIMS65	0.691(0.651–0.731)/ 0.657(0.615–0.700)	53.33(46.8–59.8)/ 37.50(29.8–45.7)	82.07(77.2–86.3)/ 93.40(89.2–96.3)	0.25
GBS	0.746(0.704–0.787)/ 0.802(0.758–0.846)	82.50(77.1–87.1)/ 64.47(56.3–72.1)	56.55(50.6–62.3)/ 78.77(72.6–84.1)	0.07
MAP(ASH)	0.769(0.731–0.806)/ 0.810(0.767–0.854)	86.67(81.7–90.7)/ 79.61(72.3–85.7)	57.24(51.3–63.0)/ 73.58(67.1–79.4)	0.16
pRS	0.623(0.578–0.669)/ 0.699(0.646–0.752)	56.67(50.1–63.0)/ 44.74(36.7–53.0)	65.52(59.7–71.0)/ 85.85(80.4–90.2)	< 0.05
T-score	0.732(0.690–0.773)/ 0.786(0.740–0.833)	71.67(65.5–77.3)/ 86.18(79.7–91.2)	66.90(61.2–72.3)/ 57.08(50.1–63.8)	0.09

#### Rebleeding

**Fig. 3:**
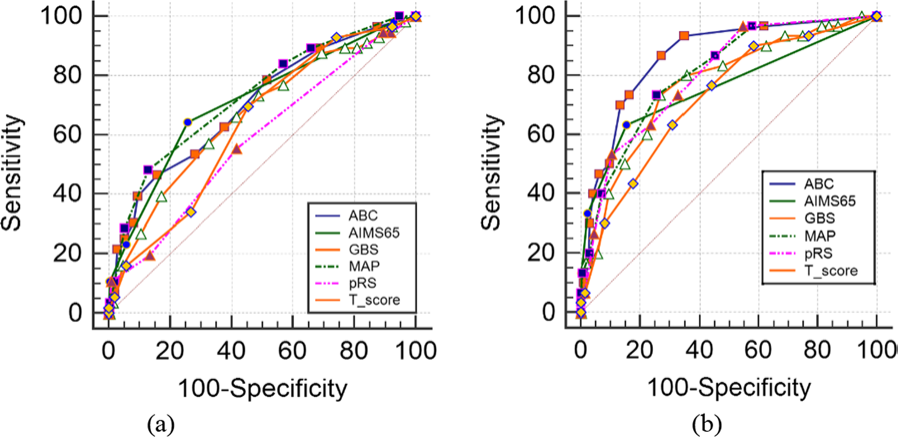
ROC curves for six scoring systems in evaluation of rebleeding. **a** Elderly group **b** younger group

The comparisons of the six scoring systems for the prediction of rebleeding are displayed in Table 5. All six systems had larger AUCs and were more effective for predicting rebleeding in the younger group. Except for ABC (p < 0.01) and pRS (p < 0.01), the differences for evaluation of rebleeding between the two groups according to the other four systems were insignificant (p > 0.05). The ROC curves for the two groups for the prediction of rebleeding are shown in Fig. 3a, b, respectively.

**Table 5:** Comparisons of ROC curves for six scoring systems in the prediction of rebleeding between the two groups

Scoring systems	AUC (95%CI)	Sensitivity %(95%CI)	Specificity %(95%CI)	P-value
Elderly group/ younger group				
ABC	0.703(0.647–0.758)/ 0.864(0.799–0.928)	46.63(37.0–56.1)/ 86.67(69.3–96.2)	84.21(80.4–87.6)/ 72.75(67.6–77.5)	< 0.01
AIMS65	0.713(0.661–0.764)/ 0.758(0.663–0.853)	64.29(54.7–73.1)/ 63.33(43.9–80.1)	74.16(69.7–78.3)/ 84.43(80.1–88.1)	0.41
GBS	0.664(0.607–0.722)/ 0.768(0.679–0.858)	57.14(47.4–66.5)/ 73.33(54.1–87.7)	67.46(62.7–71.9)/ 73.05(68.0–77.7)	0.06
MAP(ASH)	0.731(0.680–0.782)/ 0.808(0.735–0.882)	48.21(38.7–57.9)/ 73.33(54.1–87.7)	87.08(83.5–90.1)/ 74.25(69.2–78.9)	0.09
pRS	0.586(0.530–0.642)/ 0.800(0.725–0.875)	55.36(45.7–64.8)/ 53.33(34.3–71.7)	58.37(53.5–63.1)/ 89.52(85.7–92.6)	< 0.01
T-score	0.635(0.581–0.688)/ 0.723(0.630–0.815)	69.64(60.2–78.0)/ 76.67(57.7–90.1)	54.55(49.6–59.4)/ 55.99(50.5–61.4)	0.1

#### ICU admission

**Fig.4:**
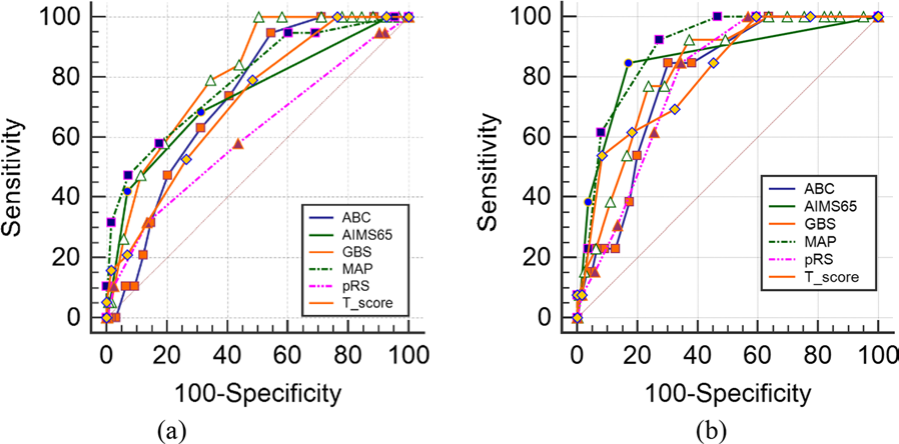
ROC curves for six scoring systems in evaluation of ICU admission. **a** Elderly group **b** younger group

The comparisons of the six scoring systems for the prediction of ICU admission are displayed in Table 6. All six systems had larger AUCs for the younger group. The AUCs of MAP(ASH) and pRS for the younger group were significantly greater than for the elderly group (p < 0.05 and p < 0.01, respectively), while the differences between the two groups according to the other four systems were insignificant. The ROC curves for the two groups can be found in Fig. 4a, b.

**Table 6:** Comparisons of ROC curves for six scoring systems in the prediction of ICU admission between the two groups

Scoring systems	AUC (95%CI)	Sensitivity %(95%CI)	Specificity %(95%CI)	P-value
Elderly group/younger group				
ABC	0.730(0.669–0.791)/ 0.786(0.696–0.876)	94.74(82.3–99.4)/ 84.62(54.6–98.1)	45.53(41.1–50.0)/ 69.80(64.7–74.6)	0.31
AIMS65	0.737(0.654–0.821)/ 0.854(0.744–0.964)	68.42(51.3–82.5)/ 84.62(54.6–98.1)	68.70(64.4–72.8)/ 82.91(78.6–86.7)	0.09
GBS	0.806(0.750–0.862)/ 0.819(0.731–0.908)	100(90.7–100.0)/ 92.31(64.0–99.8)	49.59(45.1–54.1)/ 62.68(57.4–67.8)	0.4
MAP(ASH)	0.791(0.718–0.865)/ 0.891(0.831–0.950)	57.89(40.8–73.7)/ 92.31(64.0–99.8)	82.52(78.9–85.8)/ 72.65(67.7–77.2)	< 0.05
pRS	0.610(0.514–0.706)/ 0.782(0.699–0.865)	31.58(17.5–48.7)/ 61.54(31.6–86.1)	86.59(83.3–89.5)/ 74.36(69.5–78.8)	< 0.01
T-score	0.714(0.641–0.788)/ 0.807(0.697–0.916)	78.95(62.7–90.4)/ 53.85(25.1–80.8)	51.63(47.1–56.1)/ 91.74(88.3–94.4)	0.16

For the elderly group (Table 7), in the prediction of mortality, the AUCs for ABC and MAP(ASH) were significantly higher than that of the T-score (p < 0.01 and p < 0.05). ABC was more effective than pRS. In terms of predictive intervention, pRS was worse than the other five scoring systems. MAP(ASH) performed the best and was significantly better than AIMS65. For the prediction of rebleeding, the differences between MAP(ASH), AIMS65, ABC, and GBS were not significant (p > 0.05). MAP(ASH) and AIMS65 were more effective than T-score (p < 0.05). The accuracy of pRS in the assessment of the possibility of rebleeding was significantly lower than MAP(ASH), AIMS65, and ABC (p < 0.01). The differences between GBS, T-score, and pRS were insignificant (p > 0.05). For ICU admission, MAP(ASH), GBS, T-score, ABC, and AIMS65 were similarly accurate (p > 0.05). Except for T-score, all other four scores were significantly higher than pRS (p < 0.05 for AIMS65 and ABC, and p < 0.01 for GBS and MAP).

**Table 7:** Comparison of ABC, AIMS65, GBS, MSP(ASH), pRS, T-score with significant clinical endpoints in aged group

Elderly group	AUC	P-value of pairwise the AUC curves					
		ABC	AIMS65	GBS	MAP(ASH)	pRS	T-score
Mortality							
ABC	0.827	*	0.218	0.205	0.506	** < 0.05**	** < 0.01**
AIMS65	0.762	0.218	*	0.910	0.546	0.360	0.101
GBS	0.787	0.205	0.910	*	0.579	0.256	0.052
MAP(ASH)	0.795	0.506	0.546	0.579	*	0.106	** < 0.05**
pRS	0.710	** < 0.05**	0.360	0.256	0.106	*	0.461
T-score	0.670	** < 0.01**	0.101	0.052	** < 0.05**	0.461	*
Intervetion		ABC	AIMS65	GBS	MAP(ASH)	pRS	T-score
ABC	0.713	*	0.465	0.282	0.055	** < 0.01**	0.535
AIMS65	0.691	0.465	*	0.061	< 0.01	** < 0.05**	0,161
GBS	0.746	0.282	0.061	*	0.419	** < 0.01**	0.640
MAP(ASH)	0.769	0.055	** < 0.01**	0.419	*	** < 0.01**	0.193
pRS	0.623	** < 0.01**	** < 0.05**	** < 0.01**	** < 0.01**	*	** < 0.01**
T-score	0.723	0.535	0,161	0.640	0.193	** < 0.01**	*
Rebleeding		ABC	AIMS65	GBS	MAP(ASH)	pRS	T-score
ABC	0.703	*	0.795	0.339	0.467	** < 0.01**	0.083
AIMS65	0.713	0.795	*	0.213	0.626	** < 0.01**	** < 0.05**
GBS	0.664	0.339	0.213	*	0.088	0.058	0.469
MAP(ASH)	0.731	0.467	0.626	0.088	*	** < 0.01**	** < 0.05**
pRS	0.586	** < 0.01**	** < 0.01**	0.058	** < 0.01**	*	0.216
T-score	0.635	0.083	** < 0.05**	0.469	** < 0.05**	0.216	*
ICU		ABC	AIMS65	GBS	MAP(ASH)	pRS	T-score
ABC	0.730	*	0.894	0.071	0.210	** < 0.05**	0.741
AIMS65	0.737	0.894	*	0.178	0.342	** < 0.05**	0.685
GBS	0.806	0.071	0.178	*	0.751	** < 0.01**	0.051
MAP(ASH)	0.791	0.210	0.342	0.751	*	** < 0.01**	0.147
pRS	0.610	** < 0.05**	** < 0.05**	** < 0.01**	** < 0.01**	*	0.091
T-score	0.714	0.741	0.685	0.051	0.147	0.091	*

Bold values indicate two scoring systems are statistically different from each other

*means that there is no need to compare the same scoring system

For the younger group (Table 8), in the prediction of mortality, the AUCs of ABC and pRS were significantly higher than that of GBS and T-score. In the prediction of intervention, MAP(ASH) and GBS were significantly more effective than ABC, AIMS65, and pRS, while T-score was better than AIMS65 and pRS (p < 0.01). For the prediction of rebleeding, only ABC and T-score were significantly different in terms of effectiveness. For prediction of ICU admission, only MAP(ASH) was significantly better than pRS.

**Table 8:** Comparison of ABC, AIMS65, GBS, MSP(ASH), pRS, T-score with significant clinical endpoints in younger group

Younger group	AUC	P-value of pairwise the AUC curves					
		ABC	AIMS65	GBS	MAP(ASH)	pRS	T-score
Mortality							
ABC	0.958	*	0.133	** < 0.01**	0.091	0.123	** < 0.01**
AIMS65	0.862	0.133	*	0.157	0.972	0.447	0.221
GBS	0.737	** < 0.01**	0.157	*	0.149	** < 0.01**	0.897
MAP(ASH)	0.859	0.091	0.972	0.149	*	0.384	0.215
pRS	0.913	0.123	0.447	** < 0.01**	0.384	*	** < 0.05**
T-score	0.749	** < 0.01**	0.221	0.897	0.215	** < 0.05**	*
Intervetion		ABC	AIMS65	GBS	MAP(ASH)	pRS	T-score
ABC	0.725	*	** < 0.05**	** < 0.05**	** < 0.05**	0.495	0.088
AIMS65	0.657	** < 0.05**	*	** < 0.01**	** < 0.01**	0.225	** < 0.01**
GBS	0.802	** < 0.05**	** < 0.01**	*	0.801	** < 0.01**	0.625
MAP(ASH)	0.810	** < 0.05**	** < 0.01**	0.801	*	** < 0.01**	0.461
pRS	0.699	0.495	0.225	** < 0.01**	** < 0.01**	*	** < 0.01**
T-score	0.786	0.088	** < 0.01**	0.625	0.461	** < 0.01**	*
Rebleeding		ABC	AIMS65	GBS	MAP(ASH)	pRS	T-score
ABC	0.864	*	0.071	0.088	0.264	0.204	** < 0.05**
AIMS65	0.758	0.071	*	0.881	0.416	0.496	0.606
GBS	0.768	0.088	0.881	*	0.499	0.590	0.493
MAP(ASH)	0.808	0.264	0.416	0.499	*	0.881	0.159
pRS	0.800	0.204	0.496	0.590	0.881	*	0.204
T-score	0.723	** < 0.05**	0.606	0.493	0.159	0.204	*
ICU		ABC	AIMS65	GBS	MAP(ASH)	pRS	T-score
ABC	0.786	*	0.348	0.609	0.057	0.949	0.772
AIMS65	0.854	0.348	*	0.627	0.561	0.305	0.552
GBS	0.819	0.609	0.627	*	0.187	0.551	0.867
MAP(ASH)	0.891	0.057	0.561	0.187	*	** < 0.05**	0.186
pRS	0.782	0.949	0.305	0.551	** < 0.05**	*	0.721
T-score	0.807	0.772	0.552	0.867	0.186	0.721	*

Bold values indicate two scoring systems are statistically different from each other

*means that there is no need to compare the same scoring system

## Discussion

The incidence of UGIB has declined dramatically over the past decade [19]. Nevertheless, it is among the most common and severe diseases, carrying a mortality rate of 4–10% worldwide [19, 20] and 4–14% in China [22]. In the present study, the overall mortality rate was about 5.4%, and the mortality was significantly higher in the elderly group than in the younger group (p < 0.01). Rebleeding was also significantly different between the groups. There were significant differences between the elderly and control groups in comorbidities. Elderly patients tended to have multiple complications. Comorbidities substantially impact mortality; therefore, specific attention is necessary for elderly UGIB patients [22, 23]. The general condition of older adults tends to be poor, and sometimes they can not tolerate endoscopy. Therefore, it is critical to establish scoring systems independent of endoscopy to predict outcomes of elderly patients with UGIB.

The ABC score is a newly published pre-endoscopy risk score based on age, comorbidities, and blood tests [14]. ABC accurately predicted mortality in UGIB for both groups and was superior to other UGIB scores (Tables 3, 4, 5, 6), a similar result to that reported by Laursen et al. [14]. However, ABC for elderly patients was less helpful than for younger patients. For the prediction of mortality and rebleeding, the differences were significant. This difference probably occurs because ABC considers the complications that often affect outcomes in young people, including malignant tumor and cirrhosis, but does not consider common complications in the elderly such as coronary heart disease, chronic obstructive pulmonary disease, and others that may affect outcomes. In terms of predicting intervention, ICU admission, and rebleeding for the elderly group, although ABC was not the best performing scoring system, there were no significant differences between ABC and the other four scores (except for pRS) in each item.

AIMS65 is a simple scoring system [24]. However, there are conflicting conclusions about its predictive ability [25]. In the present, AIMS65 was moderately accurate, and there was no significant difference in its use between the groups. The elderly group outperformed the younger group in predicting interventions (Table 4), the only case in all comparisons. It is not recommended to use the AIMS65 score to grade the risk of rebleeding and other aspects in ANVUGIB patients [16]; therefore, applying the AIMS65 scoring system needs further research.

GBS is the most widely used UGIB scoring system with several years of practice and is recommended by many guidelines [16]. In our research cohort, GBS showed the best ability to predict the need for ICU admission for elderly patients (Table 6). Except for rebleeding (poor accuracy for the elderly group and moderate accuracy for the younger group), GBS showed acceptable performances for both groups (Tables 3, 4, 5), which is like the results reported by Kim et al. [26]. As mentioned in the Asian-Pacific Consensus Group guideline 2018 [17], it is challenging for GBS to predict rebleeding accurately. Considering the significant difference in rebleeding between the elderly and the young populations (Table 1), attention should be paid to evaluating the elderly using GBS.

The MAP(ASH) score was established in 2020 [13]. It is a pre-endoscopic risk score for predicting intervention of UGIB and can predict the risk of death (Table 3). MAP(ASH) showed good predictive accuracy for intervention and was fair for mortality [13]. The ability to predict rebleeding is similar to GBS but superior to AIMS65. In the present study, among the six scoring systems, MAP(ASH) had the highest accuracy in predicting intervention and rebleeding and had the second-highest accuracy in predicting death and the need for ICU admission for the elderly group. More accurate performances were found in the younger group; however, there was no significant difference. MAP(ASH) was superior to the two commonly used scores (GBS and AIMS65) (Tables 3, 4, 5, 6). Nevertheless, MAP(ASH) requires validation in several clinical studies as a new score.

The pRS simplifies the Rockall score and is used for the pre-endoscopic evaluation of UGIB patients. The accuracy and applicability of the score remain controversial in clinical practice [12]. In the present study, pRS was the worst of the six scores for predicting intervention, rebleeding, and ICU admission for elderly patients. However, even for predicting mortality, it was only better than T-score. The pRS performed well in predicting mortality for the younger group and was the only scoring system with differences in all four evaluations between the groups. This finding may be related to the higher incidence of tachycardia and shock in younger patients [27]. Because the recently updated 2019 international Consensus Group guidelines did not explicitly recommend or object to the assessment of patients with very low risk of rebleeding or death based on the pRS scores [16], and the use of pRS score in the evaluation of elderly UGIB patients may not be appropriate.

T-score was proposed in 2008 to evaluate the timing of endoscopic examination in patients with UGIB [28]. In a prospective multi-center validation study, the accuracy of the T-score in predicting the risk of early endoscopy, rebleeding, and death was similar to GBS [29]. For the two cohorts in the present study, T-score performed poorly in predicting mortality and rebleeding. For predicting intervention, T-score was better than ABC, AIMS65, and pRS; however, no significant difference was observed between the groups. At present, there are few verifications of this score and a lack of solid evidence for its clinical application [30]. Further verification is necessary.

The limitations of this paper are as follows: (1) This was a retrospective study of a single-center study; (2) Patients who did not undergo esophagogastric duodenoscopy were excluded in this study, which may affect the results; and (3) Parameters such as rehospitalization and prolonged hospitalization were not analyzed. Further study is necessary.

In conclusion, elderly UGIB patients are more likely to develop severe disease and die in the hospital. More attention, appropriate triage, and early prevention should be provided to these patients. For mortality prediction, ABC had the best accuracy for both groups. However, there were significant differences between groups. Similarly, ABC had the best rebleeding prediction accuracy in the younger group and was significantly better than the elderly group. The accuracy for intervention and ICU admission was moderate, and no differences between groups were found. MAP performed fairly in all kinds of evaluations. It was the most accurate for predicting intervention in both groups. For ICU admission prediction, the younger group was significantly more effective than in the elderly group. When discussing rebleeding, GBS performed poorly in the older group. For the elderly, pRS is the worst in most cases, and all evaluation results were different from the younger group. Therefore, MAP is not suitable for evaluation in elderly patients with UGIB. Currently, no system is perfect. These systems need to be optimized, especially for elderly patients in the future.

## Data Availability

The datasets generated and/or analysed during the current study are not publicly available due [Belong to Zhongda Hospital] but are available from the corresponding author on reasonable request.

## Abbreviations

UGIB: Upper gastrointestinal bleeding
GBS: Glasgow Blatchford score
pRS: Pre-endoscopic Rockall score
AUCs: Area under the receiver operator characteristics curves
